# Supplementary material for: Prevalence of SARS-CoV-2 infection in Baja California, Mexico: Findings from a community-based survey in February 2021 in the Mexico-United States border
Source: PLOS Glob Public Health. 2022 Aug 2;2(8):e0000820. doi: 10.1371/journal.pgph.0000820 (PMC10021449; doi:10.1371/journal.pgph.0000820)
Supplement: Attachment — Submitted filename: Response to reviewers.docx [file pgph.0000820.s001.docx]

May 27^rd^, 2022

Dear editor,

Thank you so much for reviewing out manuscript titled “Prevalence of SARS-CoV-2 infection in Baja California, Mexico: Findings from a community-based survey in February 2021”.

The comments and suggestions from the editor and both reviewers are very valuable and we addressed every one of them at the best of our ability as shown below.

Please let me know if you have any other suggestions.

Kindest regards,

Oscar E. Zazueta

Principal Investigator

Journal Requirements:

**“1. Your co-authors, J. Oggun Cano-Torres (**[**oggunc@gmail.com**](mailto:oggunc@gmail.com)**), César A Méndez-Lizárraga (**[**melc.cesar@gmail.com**](mailto:melc.cesar@gmail.com)**), Timothy C Rodwell (**[**trodwell@health.ucsd.edu**](mailto:trodwell@health.ucsd.edu)**), Neiba G. Yee (**[**neiba.yeemz@gmail.com**](mailto:neiba.yeemz@gmail.com)**), Idanya Rubí Serafín-Higuera (**[**serafin.idanya@uabc.edu.mx**](mailto:serafin.idanya@uabc.edu.mx)**), Susana González-Reyes (**[**susana.gonzalez.reyes@uabc.edu.mx**](mailto:susana.gonzalez.reyes@uabc.edu.mx)**), Jesus Rene Machado-Contreras (**[**rene.machado@uabc.edu.mx**](mailto:rene.machado@uabc.edu.mx)**), Lucy E Horton (**[**lhorton@health.ucsd.edu**](mailto:lhorton@health.ucsd.edu)**), Steffanie A Strathdee (**[**sstrathdee@health.ucsd.edu**](mailto:sstrathdee@health.ucsd.edu)**), Ruth Rodríguez (**[**beatriz@colef.mx**](mailto:beatriz@colef.mx)**), and Ietza Bojórquez-Chapela (**[**ietzabch@colef.mx**](mailto:ietzabch@colef.mx)**), have not confirmed authorship of the manuscript. We have resent them the authorship confirmation email; however please check that the above email address for them is correct and follow up personally to ensure they confirm. Please note that we cannot pass your manuscript to Production until we have received confirmations from all co-authors.**

**Just in case your co-authors are having difficulty confirming their authorship, you may advise them to send us an email at** [**globalpubhealth@plos.org**](mailto:globalpubhealth@plos.org) **and we will confirm their authorship on the authors' behalf.”**

Response: Thank you for notifying me. All authors were contacted individually and they all reported confirming authorship of the manuscript.

**“2. Please update your Competing Interests statement. If you have no competing interests to declare, please state: The authors have declared that no competing interests exist.”**

Response: In the initial submission we entered the following text in the “Competing interests” section of the “Additional Information” tab: “The authors declare that they have no known competing financial interests or personal relationships that could have appeared to influence the work reported in this paper”. Given the suggestion from the editor, we have changed it to “The authors have declared that no competing interests exist”.

**“3. In the online submission form, you indicated that your data will be submitted to the Dryad database upon acceptance. Should your submission be accepted, we will require the following information in your Data Availability Statement:**

**1. The DOI provided by Dryad
2. The citation for your data package in the reference section of your manuscript
3. The citation for your data package in the methods section**

**If you are unable to adhere to our open data policy, please kindly revise your statement to explain your reasoning and we will seek the editor's input on an exemption. Please be assured that, once you have provided your new statement, the assessment of your exemption will not hold up the peer review process."**

Response: We decided to follow the suggestion from Reviewer 1, and the dataset and its key were sent to Dryad for publication. The dataset is currently under curation and is expected to be published soon.

1. The DOI that they provided is: https://doi.org/10.5061/dryad.547d7wmbk, however, we were advised that this DOI will be active after publication. In the meantime, they provided the following provisional URL for consultation:

https://datadryad.org/stash/share/YoBLyrh74KFdLNJpoPMNSGC-pp5oUgKQXgOT6Z47heE

2. We added a reference for the data package in corresponding section (Reference No. 13): “Zazueta, Oscar E (2022). COVID-19 survey in Baja California, Mexico, in February of 2021, Dryad, Dataset, https://doi.org/10.5061/dryad.547d7wmbk”

3. The data package was cited also in the Methods section, in the subsection “Survey tool and materials”: “The questionnaire and the dataset for this survey is published and is open for consultation [13].”

The “Data availability” section on the “Additional Information” tab was updated to reflect the publication of our data package.

**“4. We noticed that you used “data not shown” in the manuscript. We do not allow these references, as the PLOS data access policy requires that all data be either published with the manuscript or made available in a publicly accessible database. Please either remove these references, or amend the supplementary material to include the referenced data.”**

Response: We removed the “data not shown” references from the text, given that all data is publicly available through Dryad. What we initially meant is that this information was not shown in figures or tables. However, the information is actually shown in the text in the Results section, plus, the data is available publicly.

**“5. We do not publish any copyright or trademark symbols that usually accompany proprietary names, eg (R), (C), or TM  (e.g. next to drug or reagent names). Therefore please remove all instances of trademark/copyright symbols throughout the text, including KoboToolBox® on page 6.”**

Response: We removed all copyright or trademark symbols. Also, we did not feel the need to mention KoboToolBox altogether, so we just mentioned that a software was used to systematize capturing data.

**“Additional Editor Comments (if provided):**

**Seroprevalence surveys provide an important snapshot during the COVID pandemic. The pandemic moves quickly so prevalence data and vaccination rates change quickly. I would in particular separate out the information on vaccine. Perhaps remove vaccination status from table 1 and have a separate section discussing that in light of roll out and policy stage at time vaccination rates were low and 'pre-contemplative'. I might provide more info on movement and travel restrictions as well as vaccine roll out at the time, as the early phase of COVID may not be remembered in the future when the paper is read later on.”**
Response: We appreciate your suggestion. Both reviewers also suggested to explain in more detail the national and state vaccination campaigns and its status at the time of the survey. In light of those valuable suggestions, we explained it in more detail in the introduction section. Also, since the number of people vaccinated was too low, the information from vaccination status was removed from the models (now shown in Table 5) as Reviewer 1 suggested.

**“Reviewer #1: The authors carried out a cross-sectional study to evaluate COVID-19 prevalence the state of Baja California, Mexico. The paper is interesting, but there are some pieces of information that should be added to help the interpretation of results. Some specific comments follow:

Lines 49-41: the conclusions of the abstract seem quite different from those of the paper, and not fully supported by the results. The authors should align them.”**
Response: We thank you for the important remark. We edited the final part of the abstract in order to have conclusions that are more aligned with the results and discussion of the manuscript.

**“Line 95: how long was the survey period? It seems unlikely that all subjects were tested in only one day (1-19-2021)”**
Response: We apologize for the confusion, we meant to point out that the survey was conducted between February 1^st^ and February 19^th^ of 2021. Thank you for pointing it out. The “Methods” section was edited to make this clear now.

**“Lines 185-186. It is difficult for the reader to interpret the reported prevalence of vaccinated subjects (<1%) without putting this results in the context of the vaccination policy of Mexico. Some basic information about it should be provided in the introduction section. As national campaigns of immunization started in many countries at the beginning of 2021, the observed result could be not necessarily worrying.”**
Response: Thank you for pointing this out. We included a description of the national vaccination campaign in the “Introduction” section of the manuscript, as well as a line in the “results” section putting this specific result in context.

**“Table 1. row percentages would have been much more informative than column ones. If the Authors report, for example, that seroprevalence was 10% in men and 15% in women, this is something that can be directly interpreted. On the contrary, the reported percentage do not have a clear interpretation. Moreover, the sum of these percentages sometimes is more than 100% (probably due to rounding issues). Please double-check them.”**
Response: Thank you for the suggestion. Based on its relevance, we followed the suggestion and decided to present a new table. Table 1 now shows general characteristics of the patients and Table 2 presents the seropositivity with row percentages to illustrate the important point that the reviewer makes.

**“Line 227. One of the most striking results is the much higher RT-PCR prevalence of SARS-CoV-2 in Ensenada (22.2%) compared to Tijuana (6.4%) and Mexicali (5.5). Moreover, it is notable that seroprevalence in Ensenada was 21.9, lower than RT-PCR prevalence, which seems odd, and very similar to the other two cities. How do the authors explain these apparently contrasting results? Were the official figures (cases and deaths) very different among these cities at the beginning of 2021?”**

Response: This is a very important aspect of the study that both reviewers addressed in their comments. In light of this, the revise manuscript now shows additional information comparing incidence and mortality rates during the first weeks of 2021 across the three cities in the “Discussion” section. This could explain the observed differences, although more research is needed to understand the underlying causes of this phenomenon.

**“Table 4. the estimated effect of vaccination is based on a very small number of subjects (<1% of them were vaccinated) and is thus probably unreliable. For this reason, this variable should not be included in the model.”**
Response: We followed the recommendation and deleted the vaccination status from the model. Table 5 (Table 4 in the previous version of the manuscript) now reflects those changes. Thank you for the suggestion.

**“Lines 278-279. “however, the observed differences may be explained by the study design and potential selection bias.” Some more information is required here. Is the previous or the present study that the Authors are suggesting that was affected by selection bias?”**

Response: We implied that the referenced study (Reference 18 from Muñoz-Medina JE et al.) may be affected by selection bias since their sample came from blood banks and clinical laboratories, and not from the general population. We edited this lines slightly to avoid any confusion. Thank you for pointing it out.

**“Lines 295-296 “This study had several limitations. First, a high refusal rate may have impacted the representativeness of the sample.” Again, some more information and discussion are required here, as this is a major weakness of your study. The refusal rate seems to be about 60%, thus the possibility that selection bias affected your estimates is pretty high. Is there anything the Authors can say to increase confidence in these results?”**

Response: Thank you for your valuable comment. We explored this issue in more detail in the “Discussion” section of this revised manuscript, and we point out that even though this issue was partially addressed by weighting of the results, the possibility of having selections bias cannot be discarded.

**“Lines 306-309 I assume that the main reason for border crossing restrictions was that prevalence of COVID-19 was much higher on one side than the other, not that active border crossing was itself considered a risk factor for COVID-19 or that Baja California had a higher prevalence than the rest of Mexico. The authors should clarify this point.”**
Response: Thank you for pointing this out. The introduction section addresses this point briefly: “As part of an international effort to reduce viral transmission in the border region, the U.S. reached agreements with Mexico to limit all non-essential travel across their borders starting March 20, 2020 [6].”, with reference 6 being the statement by the U.S. Department of Homeland Security. Unfortunately, the statement does not go into detail on the reasons for this agreement nor provide any data that suggests that the COVID-19 situation was worse in Baja California or other border States in Mexico, as opposed to border States in the U.S.

**“Lines 312-313 “While the use of facemasks and hand washing was frequent, adopting quarantine or home isolation as a risk reduction measure was less common…”. While facemask use and hand washing are general rules for the population, quarantine or home isolation apply in specific circumstances (e.g., being tested positive). Thus, it is not completely clear whether it is correct to compare the prevalence of these two types of measures.”**
Response: Thank you for pointing this out. At the time of the survey, the State of Baja California had just experience the second major wave of cases and deaths, and while staying at home was not mandatory, it was highly recommended by health authorities that only “essential activities” continued operations, with many sectors of the population adopting “home-office” activities in order to reduce mobility. The purpose of this question was to assess how strong people adhere to this recommendation. Perhaps we should have used the term “staying at home” instead of “Quarantine” and “home isolation”, but these were the terms used in the questionnaire to refer to staying at home as a preventive measure.

**“Data Availability
The data should be provided as part of the manuscript or its supporting information or deposited to a public repository duing the submission, not after.”**

Response: Thank you so much for this recommendation. We decided to upload the dataset and keys to DRYAD. The data package is currently in a curation process and will be published soon. In the meantime, reviewers can access the data package in the following link: https://datadryad.org/stash/share/YoBLyrh74KFdLNJpoPMNSGC-pp5oUgKQXgOT6Z47heE

**“Reviewer #2: This is a short-term survey of SARS-CoV2 active infection and seroprevalence in a population in CA Mexico. While the work is of a value to the local community and the country, there are some concerns with the data analyses that can be improved. Here are my comments:**

**1. First major challenge is that the study was conducted over a short period of time and with one time assessment of the population. A major limitation and should be justified.”**

Response: We thank the reviewer for this valuable comment. While most of the data used for public health decision-making comes from epidemiological surveillance systems, population-based assessments are more difficult to find since they require a probability-based active search of cases. From our point of view, the methodology employed in our study and its results show that this kind of assessments provide a very different scope of what we see in conventional epidemiological data.

On top of the methodology employed, the Baja California region is of particular interest for being a key area in the Mexico-United States border, and it is reported to being the busiest port of entry in the Unites Stated (Tijuana-San Diego border), and one of the busiest ports of entry in the world. As mentioned in the “Introduction” section of this manuscript, the travel restrictions that took place between March of 2020 and November of 2021 were applied as a preventive measure and with the goal of reducing viral transmission across the border region. We hope that the results of this study and its implications are valuable for future health policy making, and we believe that it will foster discussion in the Global Health community on the need of in-depth assessments of the effectiveness of travel restrictions as a preventive measure for infectious diseases. In light of valuable comments from both reviewers, we emphasized the importance of the area of the study in the title of manuscript and abstract.

**“2. Second major major challenge is that there are no data provided to distinguish whether seroprevalence and/or active infection is due to infection or vaccination. The authors need to include vaccination data (from the surveys on the subject) and relate them to the infection and seroprevalence status.”**
Response: We thank the reviewer for this valuable comment. We included a brief explanation on the national and state strategies for vaccination at the time of the survey. Up until February 19^th^ of 2021, the estimated coverage of people with at least one dose was less than 0.3%. In order to illustrate this point, we emphasize now in the abstract and in the introduction that this study took place before the population-wide vaccination campaign started.

**“3. Minor comments
a. why infection PCR prevalence highest in Ensenada compared to other cities?”**

Response: Thank you for pointing out this important result of our study. Given that Reviewer 1 also raised this question, we decided to elaborate on this in the discussion section of the manuscript.

**“b. Table 3. Please add no. subjects for each location.”**
Response: Thank you for the suggestion. We added a new column with the number of participants per city.

**“c. You should conduct stratification analysis by age (and co-morbidities) and smoking status in relation to PCR prevalence and seroprevalence?”**

Response: Thank you so much for the suggestion. Table 2 now explores the seroprevalences stratified by age category, as well as for comorbidities (diabetes, obesity, hypertension, cardiovascular disease, COPD, cancer, HIV/AIDS) and smoking status.

Table 4 also explores the association of these covariables with the circulation of IgG antibodies against SARS-CoV-2.
